# Impact of COVID-19 on Ocular Surface Health: Infection Mechanisms, Immune Modulation, and Inflammatory Responses

**DOI:** 10.3390/v17010068

**Published:** 2025-01-06

**Authors:** Duliurui Huang, Weixia Xuan, Zhijie Li

**Affiliations:** 1 Department of Ophthalmology, Henan Provincial People’s Hospital, People’s Hospital of Zhengzhou University, Zhengzhou 450003, China; huangduliurui99@163.com; 2 Department of Respiratory and Critical Care Medicine, Henan Provincial People’s Hospital, People’s Hospital of Zhengzhou University, Zhengzhou 450003, China; xuan_1269@163.com; 3 Henan Eye Institute, Henan Eye Hospital, Henan Provincial People’s Hospital, People’s Hospital of Henan University, People’s Hospital of Zhengzhou University, Zhengzhou 450053, China

**Keywords:** COVID-19, ocular surface immunity, inflammatory response, SARS-CoV-2, cytokine storm

## Abstract

COVID-19, caused by SARS-CoV-2, has presented formidable challenges to global health since its emergence in late 2019. While primarily known for respiratory symptoms, it can also affect the ocular surface. This review summarizes the effects of SARS-CoV-2 on ocular surface immunity and inflammation, focusing on infection mechanisms, immune responses, and clinical manifestations. Ocular symptoms, though uncommon, include conjunctivitis, dry eye, and blurred vision. SARS-CoV-2 binds to ACE2 receptors in ocular surface epithelial cells, facilitating viral entry, replication, and local dissemination. The innate immune responses involving corneal epithelial cells and immune cells are discussed, alongside mechanisms of antigen presentation and adaptive immunity. The review also examines the roles of cytokines and chemokines in mediating ocular surface inflammation and explores the impact of cytokine storms and chronic inflammation on ocular health. Additionally, the interplay between systemic and ocular immune responses is highlighted, analyzing how systemic COVID-19 inflammation influences ocular surface health. These insights underscore the broader implications of COVID-19 beyond localized ocular infection. By consolidating current findings, this review aims to guide preventive and therapeutic strategies while identifying directions for future research to mitigate the ocular consequences of COVID-19.

## 1. Introduction

COVID-19 (Coronavirus Disease 2019), caused by SARS-CoV-2, has not only posed significant challenges to global public health but also unveiled the complex impact of viral infections on multiple organ systems. As a key organ exposed to the external environment, the ocular surface serves not only as a potential entry portal for the virus but also exhibits intricate interactions with the systemic immune system [1,2]. In recent years, research on the mechanisms of SARS-CoV-2 transmission via the ocular surface, its immune and inflammatory responses, and associated clinical manifestations has grown substantially. This review aims to systematically explore the multifaceted impacts of COVID-19 on the ocular surface, including infection mechanisms, immune modulation characteristics, and clinical implications. The focus is on elucidating virus–host interactions and providing insights to guide future research and prevention strategies.

The ocular surface, comprising structures such as the conjunctiva, cornea, and lacrimal glands, serves as one of the body’s primary barriers to the external environment. It plays a pivotal role in defending against pathogen invasion and maintaining local immune homeostasis. Studies have demonstrated that SARS-CoV-2 invades cells via ACE2 receptors expressed in ocular surface tissues, exacerbating tissue damage through local and systemic inflammatory responses [3,4]. Additionally, tears, as a potential transmission medium, may act as a viral conduit and facilitate interconnection with the respiratory system through tear circulation, further complicating the dynamics of viral spread. Thus, understanding the role of the ocular surface in COVID-19 transmission and immune regulation is critical for advancing knowledge of viral infection mechanisms [5,6].

Recent advances in research have shed light on the molecular basis of the ocular surface’s role in COVID-19 transmission and its immune regulatory functions. The expression of ACE2 and TMPRSS2 proteins in corneal and conjunctival tissues provides a molecular basis for viral entry into ocular surface cells. Furthermore, SARS-CoV-2 RNA has been detected in the tears of approximately 3–7% of COVID-19 patients, supporting the hypothesis of the ocular surface as a potential transmission route [7]. These findings highlight the dual role of the ocular surface as both a portal for direct viral infection and a critical site for local immune and systemic inflammatory responses [2].

It is noteworthy that the immune responses induced by COVID-19 are not confined to localized inflammation but may also exacerbate ocular surface damage through systemic immune feedback mechanisms. Systemic immune phenomena, such as cytokine storms, further underscore the potential long-term implications of SARS-CoV-2 on ocular health. These studies not only emphasize the importance of ocular surface immune regulation but also offer new directions for optimizing prevention and treatment strategies [3,5].

## 2. COVID-19 and Ocular Symptoms

### 2.1. Epidemiological Data on Ocular Symptoms in COVID-19 Patients

During the COVID-19 pandemic, epidemiological data on ocular symptoms in patients have been gradually studied and reported. Although the overall incidence is low, the clinical significance of these symptoms warrants attention. Large-scale studies indicate that the prevalence of ocular symptoms in COVID-19 patients ranges from 1% to 5%, with some reports suggesting rates as high as 7% to 10%, reflecting significant variations across regions and patient populations [8,9]. For instance, in developing countries, the incidence of ocular symptoms may be higher due to limited public health resources, potentially related to insufficient eye protection measures [10].

Research also highlights that elderly patients, immunocompromised individuals, and those with preexisting ocular conditions, such as dry eye or conjunctivitis, are more prone to develop ocular symptoms. These patients commonly report redness, photophobia, and abnormal tear secretion, which may be linked to compromised immune regulation [11]. Specific groups, such as children and adolescents, also show distinct characteristics and prevalence of ocular symptoms. Studies suggest that children are more likely to exhibit nonspecific redness or mild conjunctivitis, while symptoms such as photophobia and eye pain are less frequently reported [12]. This disparity may be attributed to the more localized immune response of the pediatric ocular surface and differing concentrations of antiviral components in their tears [13,14].

Overall, while the general incidence of ocular symptoms in COVID-19 patients is low, their characteristics and variations in specific groups highlight the need for further research to understand the epidemiological patterns of these symptoms and to provide evidence for precise management strategies.

### 2.2. Types and Severity of Symptoms

The types and severity of ocular symptoms in COVID-19 patients vary widely, depending on the patient’s condition and systemic manifestations (Table 1). Conjunctivitis is the most commonly reported symptom, presenting as redness, itching, tearing, and increased discharge. It is typically mild to moderate, self-limiting, and of short duration [9,15]. Dry eye is another frequent complaint, characterized by dryness, foreign body sensation, and visual fatigue. This may result from COVID-19–induced tear film disruption, such as lacrimal gland dysfunction leading to reduced tear production, or instability of the lipid layer exacerbating dry eye symptoms [16,17]. The chronicity of dry eye symptoms may also be linked to persistent inflammation and immune dysregulation [18].

Another common yet often overlooked symptom is photophobia, presenting as light sensitivity, tearing, and eye pain. This could involve local inflammation–induced sensitization of trigeminal nerve innervations and the localized release of inflammatory mediators like IL-6 [8,25]. Blurred vision is also reported by some patients, often associated with corneal epithelial damage, retinal hypoxia, or choroidal inflammation, necessitating further investigation for confirmation [26,27]. Less common but more severe symptoms include eyelid edema, iritis, and uveitis. These symptoms are typically associated with severe systemic inflammation or high levels of systemic cytokine release, such as during a “cytokine storm” [28].

**Figure 1:**
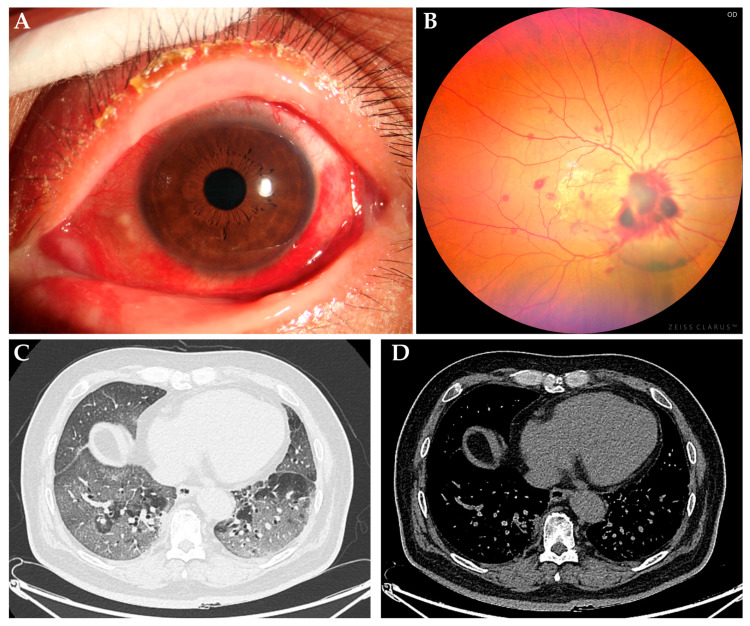
Typical cases during the epidemic. (**A**) conjunctival infection and discharge; (**B**) retinal inflammation; (**C**,**D**) bilateral lungs show multiple large, patchy, linear, and strip-like high-density opacities with blurred margins, prominently affecting the peripheral areas of both lungs.

The severity of ocular symptoms is often correlated with the patient’s overall condition. Mild cases generally involve slight discomfort, while severe cases may experience more pronounced visual impairment or elevated intraocular pressure. Comprehensive evaluation and early identification of ocular symptoms are thus crucial for the overall management of COVID-19 patients (Figure 1).

### 2.3. Case Reports and Clinical Observations

Multiple case reports and clinical observations have characterized the ocular symptoms of COVID-19 patients and their potential diagnostic value. Some reports suggest that conjunctivitis may be an early manifestation of COVID-19, with certain patients presenting unilateral or bilateral redness and increased discharge days before diagnosis [15,19,29]. This highlights the potential utility of ocular symptoms as early screening indicators for COVID-19 [9,30]. Other studies have found that ocular symptoms often coincide with or follow respiratory symptoms, suggesting that the virus may spread through tears or directly infect the ocular surface [31,32]. The successful isolation of SARS-CoV-2 from tears and conjunctival swabs using RT-PCR further supports this hypothesis. However, significant variability exists in the viral load and infectivity of tears, likely influenced by the patient’s immune status, viral strain characteristics, and disease stage [33,34]. Observations also indicate that the positivity rate for viral RNA in tears is higher in severe cases, suggesting that systemic inflammation and immune activation may facilitate local viral replication and persistence [31,35].

Although ocular symptoms are relatively uncommon in COVID-19 patients, their diversity and potential diagnostic value warrant further investigation. Systematic analysis of key patterns from case observations, such as the timing of conjunctivitis onset and its relationship with systemic symptoms, combined with global case data comparisons, can provide a more comprehensive understanding of these phenomena. These studies not only aid in the early identification and management of ocular symptoms but also offer critical insights into the transmission pathways and mechanisms of the virus.

## 3. Mechanisms of SARS-CoV-2 Infection in the Ocular Surface

### 3.1. SARS-CoV-2 and ACE2 Receptors: Expression in Ocular Surface Epithelial Cells

**Figure 2:**
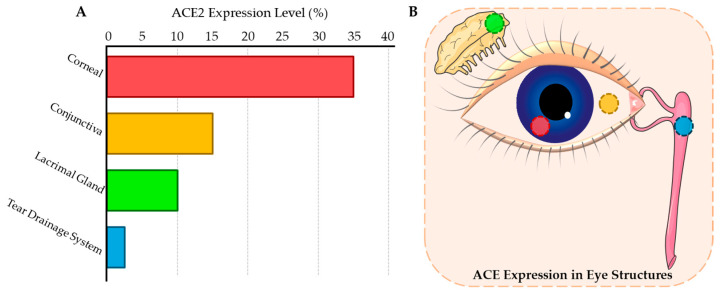
Distribution of ACE2 receptor expression in the anterior segment. (**A**) ACE2 receptor expression levels in various ocular structures were assessed. The cornea shows a high level of ACE2 expression (30–40%), while the conjunctiva exhibits moderate expression (10–20%). The lacrimal gland demonstrates low to moderate expression (5–15%), and the tear drainage system reveals relatively lower expression (1–5%). (**B**) In terms of anatomical visualization, the cornea (red), with its high ACE2 expression, serves as the primary viral entry point. The conjunctiva (yellow), exhibiting moderate ACE2 expression, represents a potential route for viral transmission and infection. The lacrimal gland (green) connects tear production with potential, though limited, respiratory transmission. Similarly, the tear drainage system (blue) contributes to the potential for respiratory transmission, albeit with lower ACE2 expression.

SARS-CoV-2 invades host cells by binding to angiotensin-converting enzyme 2 (ACE2) receptors on the cell surface. Studies have demonstrated ACE2 receptor expression in ocular surface tissues, including the conjunctiva [4,36], corneal epithelial cells [1,2,37,38], lacrimal glands [39,40,41], and lacrimal drainage system [42] (Figure 2). This distribution provides a molecular basis for viral infection via the ocular surface route [1,6]. Immunohistochemical studies specifically indicate that ACE2 expression is highest at the corneal limbus, while lower levels are observed in the conjunctiva and lacrimal gland tissues. The co-expression of TMPRSS2 (transmembrane serine protease 2), which facilitates the cleavage of the SARS-CoV-2 spike protein, further enhances viral entry capacity [4].

Notably, the characteristics of viral variants significantly affect their binding affinity for ACE2. For example, key mutations in the receptor-binding domain (RBD) of the spike protein in the Omicron variant, such as N501Y and E484A, substantially enhance affinity for ACE2, potentially increasing infection efficiency and the risk of ocular surface transmission [43,44]. Research shows that these mutations optimize the binding interface between the RBD and ACE2 receptor by altering the charge distribution and molecular conformation, thereby enhancing viral infectivity [44,45]. Investigations into these variants underscore the importance of protecting ACE2-rich areas, such as the ocular surface and other exposed mucosa, to prevent viral entry and inform the development of targeted therapies against Omicron variants [46].

Clinical observations and animal experiments also support the potential of the ocular surface as an entry portal for SARS-CoV-2. Some COVID-19 patients present with conjunctivitis and other ocular symptoms, corroborating the virus’s ability to infect ocular surface tissues [3,47]. Furthermore, in vitro culture models show that SARS-CoV-2 can effectively replicate in ocular surface cells, indicating that the ocular surface serves not only as a portal for viral entry but also as a critical site for replication and dissemination [48].

### 3.2. Potential Pathways and Mechanisms of Viral Entry into Ocular Surface Cells

**Figure 3:**
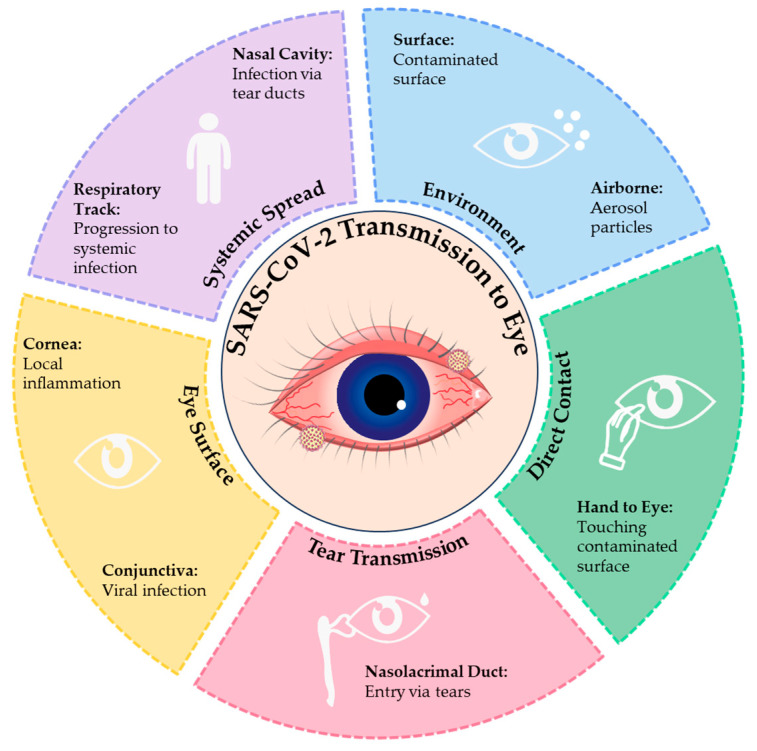
Pathways of SARS-CoV-2 transmission to the eye. This figure highlights the key routes and mechanisms of SARS-CoV-2 infection of the ocular surface: direct contact, tear transmission, and airborne spread. Direct contact occurs when contaminated hands transfer the virus to the conjunctiva or corneal epithelium. Tear transmission involves viral particles in tears reaching the ocular surface and traveling to the nasal cavity or respiratory tract via the nasolacrimal duct. Airborne spread occurs through aerosolized particles infecting the conjunctiva upon exposure. On the ocular surface, SARS-CoV-2 binds to ACE2 receptors, with TMPRSS2 facilitating spike protein cleavage and viral entry, triggering replication, local immune responses, and cytokine release. The figure underscores these interconnected transmission pathways and molecular mechanisms leading to ocular and systemic infection.

SARS-CoV-2 can infiltrate ocular surface cells through multiple pathways, including direct contact transmission, tear-mediated transmission, and aerosol exposure (Figure 3). Direct contact transmission is a substantial route, whereby the virus may infect conjunctival and corneal epithelial cells when contaminated hands touch the eyes [1,49]. Tear-mediated transmission is another key pathway. The tears of infected individuals contain viral particles, which may travel through the nasolacrimal duct to the nasopharynx, potentially enabling viral spread and subsequent infection of ocular surface tissues [2,50].

The possibility of airborne transmission, particularly via aerosol exposure, has also gained attention. Experimental simulations have demonstrated that viral aerosols can infect ocular surface cells through conjunctival tissues. Mouse model studies further reveal that aerosol exposure can induce conjunctivitis and localized inflammation [48]. These findings emphasize the potential role of airborne transmission in ocular surface infections and highlight the importance of eye protection measures.

The molecular mechanism of viral entry into ocular surface cells primarily relies on the interaction between the spike (S) protein of SARS-CoV-2 and the ACE2 receptor. Upon cleavage of the S protein by TMPRSS2, the S protein facilitates fusion between the viral envelope and the host cell membrane, allowing viral RNA to enter the host cytoplasm and initiate infection [51,52]. Furthermore, recent studies indicate that specific chemokine release by ocular surface cells may promote the dissemination of viral particles via tears, exacerbating the risk of infection [3,38].

### 3.3. Replication and Dissemination of SARS-CoV-2 in Ocular Surface Tissues

After SARS-CoV-2 enters ocular surface cells, its RNA utilizes the host cell’s transcription and translation machinery to initiate replication and generate new viral particles. The replication process involves several critical steps. Initially, the viral RNA is translated into nonstructural proteins in the host cytoplasm. These proteins assemble into the replication–transcription complex (RTC) [52,53], which replicates the viral RNA via a template mechanism, producing new genomic RNA and subgenomic mRNA [54]. Subsequently, newly synthesized viral structural proteins are assembled into viral particles within the endoplasmic reticulum and Golgi apparatus, which are then released extracellularly through a budding mechanism [55]. The replication of the virus triggers a local inflammatory response, leading to the release of significant amounts of cytokines (e.g., IL-6, IL-1β) and chemokines. These inflammatory mediators act via autocrine and paracrine pathways to recruit immune cells to the site of infection, potentially exacerbating tissue damage. Studies indicate that in the early stages of infection, corneal and conjunctival cells release interferons (e.g., IFN-α and IFN-β), which inhibit viral spread. However, excessive inflammation may compromise the corneal epithelial barrier and result in chronic inflammation.

Tear-mediated transmission plays a crucial role in the dissemination of the virus within ocular surface tissues. Viral particles in tears may travel through the nasolacrimal duct to the nasal cavity and upper respiratory tract, thereby contributing to systemic infection [1,56]. Additionally, variations in viral load within the tears of individual patients may reflect the severity of the disease and the intensity of systemic inflammatory responses [57].

In summary, the replication and dissemination of SARS-CoV-2 in ocular surface tissues not only substantially affect local tissue health but also may serve as a medium for further transmission via tears or aerosols. Understanding these mechanisms is critical for developing effective protective strategies and targeted therapeutic approaches.

## 4. Immune Responses of the Ocular Surface

**Figure 4:**
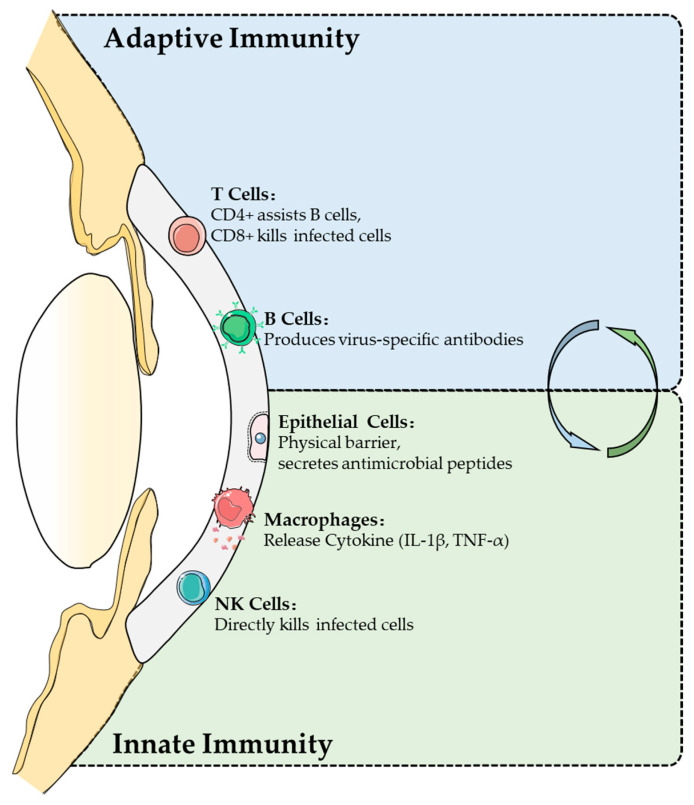
Comparative overview of innate and adaptive immunity on the ocular surface. This figure highlights the synergistic interaction between innate and adaptive immunity on the ocular surface. Innate immunity provides rapid defense through physical barriers, such as epithelial cells, which secrete antimicrobial peptides (e.g., β-defensins). Macrophages phagocytose pathogens and release cytokines (e.g., IL-1β, TNF-α), while natural killer (NK) cells directly eliminate infected cells. Adaptive immunity, in contrast, ensures long-term protection through T and B cells. CD4+ T cells assist B cells in antibody production, CD8+ T cells directly kill infected cells, and B cells differentiate into plasma cells to produce virus-specific antibodies. Arrows illustrate how innate immunity, via mechanisms such as macrophage activation, facilitates the initiation of adaptive immunity, while T cells support B cells in generating antibodies. This figure underscores the collaborative role of both immune mechanisms in combating ocular surface infections, including those caused by SARS-CoV-2.

The ocular surface innate immune system serves as the first line of defense against SARS-CoV-2, relying on corneal epithelial cells, macrophages, neutrophils, dendritic cells, and natural killer (NK) cells to mount a rapid antiviral response [58,59,60] (Figure 4). Corneal epithelial cells, in particular, form a physical barrier through tight junctions and secrete antiviral factors such as β-defensins and lysozyme, which disrupt viral membranes and help regulate immune cell migration. These cells also detect pathogen-associated molecular patterns (PAMPs) via pathways including NF-κB and IRF3, thus promoting the expression of interferons and additional antiviral genes [61,62]. As the infection progresses, macrophages and neutrophils migrate quickly to the site of infection, phagocytize pathogens, and release inflammatory cytokines (e.g., IL-1 and TNF-α) to amplify local defenses [63]. Concurrently, dendritic cells capture viral antigens and travel to regional lymph nodes, while NK cells directly eliminate virus-infected cells [64]. Taken together, these innate mechanisms are pivotal in containing early viral spread and priming the adaptive immune response.

Building upon this foundation, the adaptive immune response at the ocular surface centers on antigen presentation by dendritic and Langerhans cells, as well as subsequent T and B cell activation [65,66]. After capturing and processing viral particles, antigen-presenting cells (APCs) display peptide fragments on major histocompatibility complex (MHC) molecules, leading to T cell activation. CD4⁺ T cells differentiate into effector subsets (e.g., Th1, Th2) and memory cells, whereas CD8⁺ cytotoxic T lymphocytes (CTLs) directly target infected cells [67,68]. B cells, receiving help from T cells, develop into plasma cells that produce neutralizing antibodies, which can block viral entry and trigger complement-mediated destruction [69,70] (Figure 4). Crucial to efficient viral clearance is the balance between Th1- and Th2-driven responses. While Th1-skewed immunity facilitates viral eradication, it may also intensify local inflammation, whereas a dominant Th2 profile can dampen inflammation but reduce antiviral efficiency [71]. This delicate equilibrium is frequently disrupted in COVID-19–related ocular inflammation, potentially exacerbating tissue damage.

Further modulating these responses are cytokines and chemokines, which regulate the intensity and duration of ocular inflammation by mediating crosstalk between innate and adaptive immune cells [72,73]. Early in the infection, corneal epithelial and resident immune cells rapidly release pro-inflammatory cytokines, such as IL-1β, TNF-α, and IFN-γ, increasing vascular permeability and recruiting additional immune cells to the site. However, the “cytokine storm” phenomenon—characterized by the excessive release of IL-6, TNF-α, and other factors—can compromise corneal and conjunctival epithelial barriers, precipitating acute pathologies like conjunctivitis and keratitis, as well as more persistent complications such as dry eye and corneal ulcers [74,75]. Targeted inhibition of IL-6 or TNF-α has therefore been proposed to mitigate ocular surface damage and preserve tissue integrity in COVID-19 patients [76]. Understanding how the immune privilege of the ocular surface adapts—or fails to adapt—to viral infection and sustained inflammation is key to devising therapeutic strategies that protect this sensitive organ without triggering pathological immune responses.

Finally, despite its robust local defense mechanisms, the eye is considered an immune-privileged organ, meaning it possesses specialized immune-regulatory processes that minimize detrimental inflammation and protect visual function [77,78]. Within the ocular surface, such privilege is maintained by factors like immune-modulatory molecules, tolerance-promoting antigen-presenting cells, and the blood–ocular barrier, all of which shape the overall immune environment. This unique immunological milieu becomes especially relevant during SARS-CoV-2 infection, as it can influence how viral particles are detected, eliminated, or tolerated.

## 5. Ocular Surface Inflammatory Responses Induced by COVID-19

### 5.1. Pathophysiological Mechanisms of Ocular Surface Inflammation

Ocular surface inflammation caused by COVID-19 arises from a multifaceted interplay between viral infection–induced cellular damage and host immune responses. SARS-CoV-2 enters ocular surface epithelial cells via ACE2 receptors, initiating apoptosis and necrosis that elicit local immune activation [79,80]. Infected epithelial cells of the cornea, conjunctiva, and lacrimal glands rapidly trigger innate immunity by releasing inflammatory mediators—such as cytokines and chemokines—which then recruit immune cells (e.g., neutrophils, macrophages, and dendritic cells) to the site of infection. This cascade produces a positive feedback loop culminating in acute inflammation that clinically manifests as hyperemia, edema, discharge, and ocular discomfort [81,82].

In addition to direct tissue infection, tears may act as a potential route for viral shedding and transmission. However, studies report variable findings regarding the presence and quantity of SARS-CoV-2 in tear fluid, with some indicating detectable viral RNA and others finding minimal or no viral load. This inconsistency may be related to differences in disease severity, timing of sample collection, and individual immune responses. Further research is needed to clarify the correlation between viral load in tears, clinical progression, and the risk of ocular or systemic spread.

Notably, COVID-19–related inflammation can extend beyond the ocular surface to involve the retina, optic nerve, and other intraocular structures, potentially causing visual impairment [83]. Moreover, the robust systemic inflammatory response often seen in severe cases can exacerbate local ocular injury, placing patients at risk for both acute pathologies and chronic conditions, including dry eye and corneal ulcers.

### 5.2. Effects of Cytokine Storm on Ocular Surface Tissues

**Figure 5:**
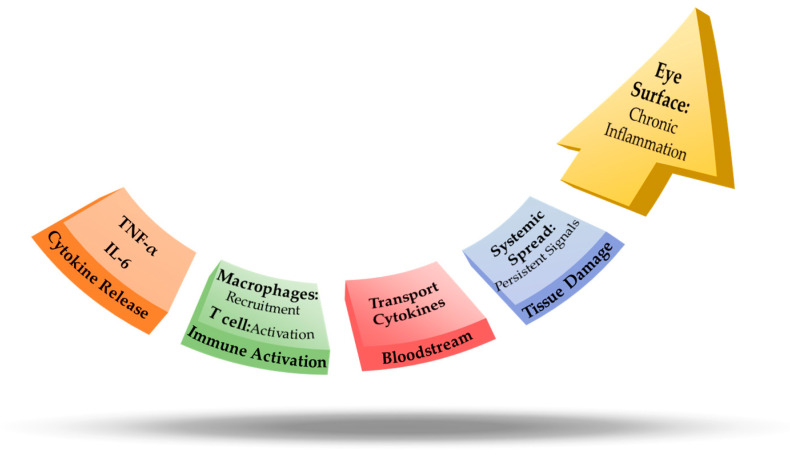
Impact of cytokine storm on ocular surface inflammation. This figure illustrates the progression of a cytokine storm, beginning with the release of pro-inflammatory cytokines, such as IL-6 and TNF-α, followed by their transport through the bloodstream. These cytokines activate immune cells, including T cells and macrophages, which intensify the inflammatory response. This immune activation results in chronic inflammation and tissue damage on the ocular surface. The localized inflammation can further propagate systemically through persistent signaling in the bloodstream, potentially leading to widespread systemic effects. The figure underscores the sequential transition from cytokine release and immune activation to chronic ocular inflammation and systemic dissemination, emphasizing the role of cytokine storms in COVID-19–related ocular surface pathophysiology.

A “cytokine storm”, frequently observed in severe COVID-19, is typified by hyperactive immune signaling and the excessive release of proinflammatory cytokines and chemokines (Figure 5). While primarily linked to pulmonary and systemic damage, these elevated cytokines—especially IL-6, IL-1β, TNF-α, and IFN-γ—can also profoundly affect the ocular surface [84,85]. Vascular dilation and increased permeability lead to tissue edema and conjunctival hyperemia, manifesting clinically as redness, swelling, and increased tearing. Excess inflammatory mediators and proteolytic enzymes may further damage corneal and conjunctival epithelial cells, weakening barrier integrity and predisposing patients to corneal ulcers, perforation, or secondary infections [86,87].

From a clinical standpoint, recognizing and managing the local effects of a cytokine storm is critical for preserving vision and minimizing ocular morbidity. While systemic anti-inflammatory therapies such as corticosteroids or IL-6 inhibitors can help control rampant inflammation, careful monitoring is essential to balance the risk of immunosuppression with the need to arrest tissue damage. Emerging strategies, including targeted inhibition of TNF-α and other key inflammatory pathways, hold promise for mitigating the ocular consequences of cytokine storms. Such targeted therapy could reduce excessive inflammation, prevent chronic sequelae, and improve long-term patient outcomes.

### 5.3. Chronic Inflammation and Long-Term Consequences

Ocular surface inflammation initiated by SARS-CoV-2 infection may persist beyond the acute phase in some individuals, transitioning into chronic inflammation. Persistent low-level viral presence, or residual viral components, can maintain local immune activation and continually stimulate inflammatory pathways [82,88]. Additionally, immune dysregulation—characterized by the sustained release of cytokines and ongoing infiltration of immune cells—can injure ocular surface tissues and impair normal repair processes.

Fibroblast activation and tissue fibrosis represent another crucial mechanism underlying chronic ocular surface changes. Excessive collagen deposition and fibrotic remodeling not only diminish corneal transparency and conjunctival elasticity but can also lead to severe and potentially irreversible visual impairment [89]. Future therapeutic interventions may include novel anti-fibrotic agents, localized immunomodulators, or combination therapies aimed at both reducing persistent inflammation and preserving tissue integrity. Ongoing research in this area will be instrumental for developing effective clinical strategies to manage the long-term ocular complications of COVID-19.

## 6. Interactions Between the Ocular Surface and Systemic Immune System

### 6.1. Connection Between the Ocular Surface and Systemic Immune Responses

**Figure 6:**
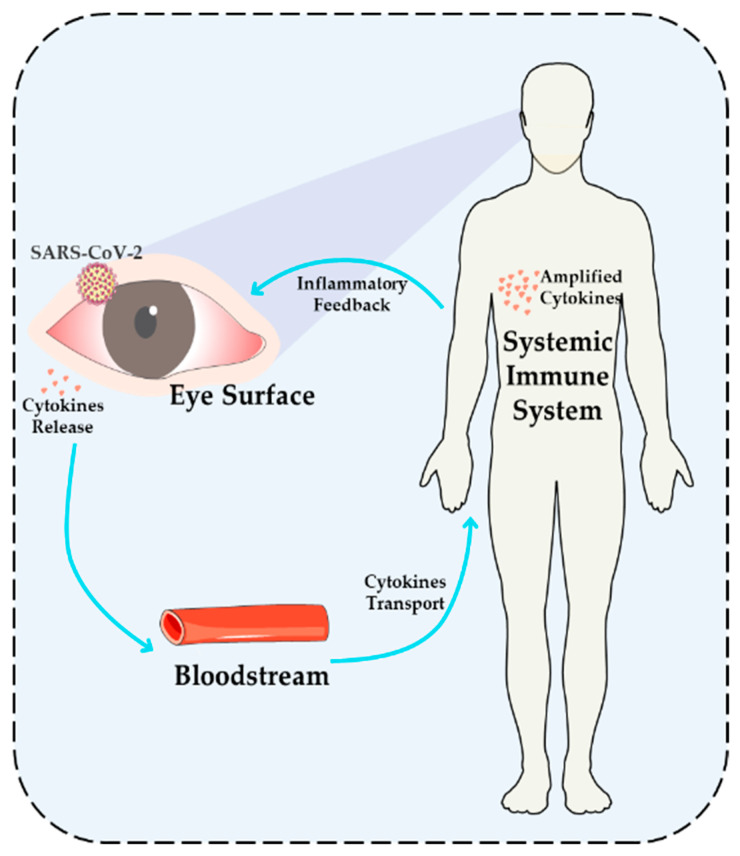
Interaction mechanism between ocular surface and systemic immune feedback. This figure illustrates the dynamic interplay between the ocular surface and the systemic immune system during viral infection. SARS-CoV-2 infection of the ocular surface triggers localized inflammation and the release of pro-inflammatory factors (e.g., IL-6 and TNF-α) into the bloodstream. These factors circulate through the vascular system, activating the systemic immune response. In turn, the systemic immune system amplifies the release of cytokines, which feedback to the ocular surface, exacerbating and perpetuating local inflammation. This vicious cycle highlights the complex impact of viral infections on both the ocular surface and systemic immunity, underscoring the need for systemic therapeutic strategies.

The ocular surface is intimately linked with systemic immunity via mechanisms such as antigen presentation, cytokine and chemokine release, and blood/lymphatic circulation (Figure 6). Antigen-presenting cells (APCs), including Langerhans and dendritic cells, can migrate to regional lymph nodes after capturing viral antigens, thus generating systemic T cell activation [79]. In parallel, corneal and conjunctival cells release cytokines and chemokines that enter systemic circulation and modulate immune responses at distant sites—for instance, corneal cell-derived IL-6 can trigger systemic acute-phase reactions [90]. Moreover, antibodies and complement factors generated in response to ocular infections disperse through vascular and lymphatic routes, bolstering overall immune defense [91]. In COVID-19, these interactions facilitate not only the local ocular response but also the spread of immune activation throughout the body, underscoring the need for integrated strategies that address both local and systemic disease processes.

### 6.2. Feedback Mechanisms of Systemic Immune Responses on the Ocular Surface

Systemic immune responses heavily influence ocular surface health through multiple feedback loops. High levels of proinflammatory cytokines, such as TNF-α, IL-1β, and IL-6, produced in severe COVID-19 can reach ocular tissues via the bloodstream, exacerbating local inflammation [92]. Similarly, an upregulation of chemokines like CXCL8 (IL-8) and CCL2 drives the recruitment of neutrophils, monocytes, and other immune cells to the ocular surface, intensifying tissue damage. Complement activation, particularly the release of C3a and C5a, further heightens local immune responses, contributing to the microvascular injury associated with advanced disease states [93,94]. The net effect of these feedback mechanisms is often persistent ocular surface damage, potentially resulting in chronic inflammation, fibrosis, and vision impairment. Mitigating such systemic-to-local propagation of inflammation is essential for reducing the risk of long-term ocular complications.

### 6.3. Impact of COVID-19–Associated Systemic Inflammation and Immune Responses on the Ocular Surface

Severe COVID-19 frequently induces a cytokine storm that can lead to widespread organ involvement, including the ocular surface. Elevated levels of IL-6, TNF-α, and IL-1β, among other cytokines, trigger intense local inflammatory responses and infiltration of immune cells (e.g., neutrophils, macrophages, and T cells) into corneal and conjunctival tissues [95,96,97]. The proteolytic enzymes and reactive oxygen species released by these cells often damage epithelial structures and weaken the ocular surface’s protective barrier.

Increased vascular permeability, a hallmark of systemic inflammation, also contributes to ocular manifestations such as conjunctival hyperemia, edema, and excessive tear production [19,98]. Chronic inflammation following COVID-19 can promote fibrosis, potentially impairing corneal clarity and conjunctival function. While systemic therapies—such as corticosteroids or immunosuppressants—help alleviate inflammation, clinicians must remain vigilant to the possibility of heightened susceptibility to secondary ocular infections. Consequently, further research into localized immunomodulatory treatments could provide safer, more targeted approaches, balancing the need to quell inflammation without broadly suppressing immune defenses [99]. A better understanding of these systemic-to-local effects is paramount in crafting comprehensive management strategies that address both acute and long-term ocular complications in COVID-19.

## 7. Current Research Limitations and Proposed Solutions

### 7.1. Limited Sample Size

Insufficient sample size remains a prominent limitation in current studies addressing the long-term impact of COVID-19 on ocular health. Such constraints diminish the reliability and generalizability of findings, especially when examining diverse populations that may exhibit varying ocular symptoms or distinct pathophysiological processes. Recent work by Salvetat and Zeppieri underscored that multicenter collaborations and standardized protocols—spanning different geographic regions and healthcare systems—are critical for capturing a more accurate spectrum of ocular manifestations and disease progression [100,101]. Their observations align with Parmar et al., who emphasized that pooling data from larger and more diverse cohorts would clarify whether variations in reported symptom prevalence reflect true epidemiological differences or result from methodological discrepancies [101].

To address these challenges, establishing multicenter collaborative networks can facilitate large-scale data collection, thus boosting representativeness and robustness. Such initiatives can highlight geographical and demographic variations in COVID-19’s ocular impact [19]. Longitudinal cohort studies with structured follow-up protocols enable researchers to trace trends and long-term ocular changes over time, providing insights into the natural course of the disease and associated mechanisms [102]. Meanwhile, global data-sharing platforms that consolidate international research not only enable comprehensive analyses of regional patterns but also foster cross-border collaboration and consistent methodological standards.

Leveraging electronic health records (EHRs) for large-scale clinical data analysis further strengthens evidence-based practice. When integrated with telemedicine, AI-based screening, machine learning, and big data analytics, EHR-driven approaches can identify nuanced trends and patterns, ultimately reshaping clinical workflows [103]. Additionally, encouraging public participation—for instance, through online tools where patients can report ocular symptoms—can swiftly expand sample sizes, providing real-world data that complement conventional clinical studies. Collectively, these strategies enhance the reliability and generalizability of research on COVID-19’s long-term ocular effects, as both Salvetat and Zeppieri and Parmar et al. advocated in their recent reviews.

### 7.2. Lack of Long-Term Studies on Ocular Surface Health

Long-term follow-up remains paramount for understanding the epidemiology, pathophysiology, and clinical management of chronic ocular complications arising from COVID-19. Although growing evidence points to SARS-CoV-2 as a possible contributor to persistent inflammation, corneal opacities, vision impairment, and other ocular conditions, extended follow-up data are sparse, making it difficult to establish the true incidence, severity, and progression of these disorders [104]. Such a gap in longitudinal information also impedes a nuanced understanding of how chronic immune dysregulation, ongoing inflammation, and fibrotic processes accumulate—or evolve—over time.

While earlier findings have noted chronic inflammation and potential vision-threatening outcomes, a more detailed exploration of additional unresolved questions is needed to advance clinical and scientific understanding. Specifically, uncertainties remain regarding (a) persistent viral presence within ocular tissues, which may perpetuate a subclinical infectious or inflammatory state, and (b) sustained immunological dysregulation, which could lead to progressive tissue damage and microvascular complications. Organizing these knowledge gaps in a concise table or figure offers a clear, visual approach to highlighting priority research directions for both clinicians and investigators.

In concert with these efforts, advanced imaging techniques and biomarker assessments can provide vital insights into the early stages of irreversible ocular damage. Optical coherence tomography angiography (OCTA), for instance, enables the real-time evaluation of microvascular alterations [105]. By systematically tracking changes in vascular integrity, immune markers, and viral persistence over extended periods, researchers can better identify risk factors, refine treatment protocols, and ultimately diminish the likelihood of lasting vision loss.

To facilitate a more structured approach in addressing unsolved research concerns, we have organized key issues related to long-term ocular consequences of COVID-19 into a concise table (Table 2). As illustrated in Table 2, clarifying persistent viral presence, sustained immunological dysregulation, and microvascular changes is critical for improving our understanding of chronic post–COVID-19 ocular conditions. Additionally, the table highlights how targeted approaches—ranging from longitudinal cohort studies to advanced imaging—could help identify individuals most at risk and enable early therapeutic interventions. Taken together, these recommendations form an actionable roadmap for researchers and clinicians seeking to mitigate long-term ocular sequelae and enhance patient care.

In conclusion, a robust, interdisciplinary strategy—combining multicenter longitudinal cohorts, targeted mechanistic studies, and accessible data visualization—is critical for moving beyond preliminary observations. Such an approach will not only illuminate how SARS-CoV-2 may induce or accelerate long-term ocular pathology but also inform the development of effective therapeutic and preventive interventions.

### 7.3. Inadequate Mechanistic Insights

Unraveling the mechanisms of SARS-CoV-2 infection and immune evasion in ocular tissues is essential for developing targeted treatments. While current evidence indicates that the virus binds to ACE2 receptors, with TMPRSS2 facilitating cleavage and membrane fusion [38], precise expression levels of these receptors across corneal, conjunctival, and lacrimal gland cells remain insufficiently characterized. Following viral entry, replication and dissemination within ocular tissues continue to be imperfectly understood, including the extent to which virions spread via tears and neighboring anatomical structures [106].

Both Salvetat and Zeppieri and Parmar et al. underscored that clarifying these mechanisms is pivotal in refining prophylactic and therapeutic strategies [100,101]. Potential solutions include investigating molecular targets (e.g., ACE2/TMPRSS2 inhibitors) to block viral entry and harnessing interferon-stimulating agents to counter immune evasion. Next-generation vaccines tailored to emerging variants could also curb viral replication within ocular tissues. Such efforts not only fill critical mechanistic gaps but also address real-world patient needs, where accurate knowledge of ocular viral pathogenesis could guide clinicians in preventing and managing severe or chronic disease sequelae.

### 7.4. Insufficient Research on Vaccine Impact

Although COVID-19 vaccines have significantly curbed disease transmission and severity, their long-term effects on ocular surface immunity remain inadequately explored. Present studies rarely extend beyond the acute postvaccination phase, leaving unanswered questions about dynamic immune responses and the frequency of vaccine-associated ocular adverse events. Parmar et al. proposed that multicenter vaccine studies, spanning various demographics, should be undertaken to evaluate vaccination outcomes, especially given reports of corneal graft rejection and other immunologic complications [100]. Longer-term follow-up could help detect subtle changes in ocular immunity and pinpoint individuals at higher risk of vaccine-related issues [107].

Establishing dedicated adverse event databases—focused specifically on ocular manifestations—would facilitate early detection of concerning patterns, thereby enabling prompt risk mitigation [108]. Enhancing public health surveillance via real-time reporting systems (e.g., VAERS) can capture a wider range of symptoms, thus providing a more comprehensive perspective on potential vaccine impacts [109]. By examining these data in conjunction with advanced research methodologies, the field can refine COVID-19 vaccination protocols and develop safer immunization strategies that account for ocular health, consistent with the recommendations in recent review articles [110].

### 7.5. Lack of Comparative Studies

Comparative investigations with other viral pathogens (e.g., adenoviruses, herpes simplex viruses) are crucial for discerning the unique pathogenic features of SARS-CoV-2 in ocular tissues. Salvetat and Zeppieri pointed out that standardized research frameworks are necessary to contextualize COVID-19–related ocular manifestations within the broader landscape of viral ophthalmology, while Parmar et al. highlighted the need for robust meta-analyses and laboratory-based experiments to refine prophylactic and treatment strategies [100]. Unified protocols that systematically evaluate clinical, immunological, and virological endpoints across multiple viral infections could reveal both shared and virus-specific mechanisms.

Such comparative approaches might include comprehensive meta-analyses of existing data to delineate overlapping and distinctive pathogenic processes. In vitro and in vivo models could then validate these findings, elucidating viral entry routes, replication kinetics, and immunopathological changes in ocular tissues. Finally, global collaborative networks that pool international data—thereby increasing sample diversity—would strengthen the statistical power and applicability of comparative research. This integrated framework has the potential to further elucidate SARS-CoV-2’s unique disease profile and optimize evidence-based management for ocular infections.

## 8. Future Research Directions

### 8.1. Unresolved Questions and Research Gaps

Despite substantial progress in understanding the impact of SARS-CoV-2 on ocular surface immunity and inflammation, several critical issues remain unresolved. The transmission pathways via the ocular surface require further clarification, including the role of tears in viral transmission, tissue susceptibility, and the relative importance of ocular transmission compared to other routes [1,57]. Post-recovery ocular immune changes are also poorly understood, as most studies focus on the acute phase of infection, leaving gaps in knowledge about how recovery affects the ocular immune system and contributes to long-term conditions like dry eye syndrome and chronic inflammation [111]. Additionally, there is a lack of systematic exploration of individual variability in immune responses based on factors such as age, gender, ethnicity, and preexisting eye conditions. The specific manifestations of cytokine storms on ocular tissues, their mechanisms of injury, and their role in the broader systemic inflammatory response remain areas that require deeper investigation [112]. Addressing these questions will tremendously enhance our understanding of SARS-CoV-2’s complex interactions with ocular immunity.

### 8.2. Emerging Technologies and Methods

Advancements in technology provide powerful tools for investigating SARS-CoV-2’s interactions with the ocular immune system. Single-cell RNA sequencing enables precise analysis of gene expression changes in various ocular cell types during infection, offering valuable insights into virus–host interactions [113]. Advanced imaging techniques, such as confocal and super-resolution microscopy, facilitate real-time observation of SARS-CoV-2’s effects on corneal and conjunctival microstructures, capturing dynamic cellular changes [114]. Stem cell–derived ocular surface organoid models serve as innovative platforms to simulate viral infection and immune responses, supporting research on invasion mechanisms and drug development [115]. Additionally, artificial intelligence and big data analytics, through the integration of patient data and ocular imaging, enhance diagnostic accuracy and help uncover potential immunological mechanisms associated with SARS-CoV-2 infection [116]. These cutting-edge methodologies are expected to substantially advance the understanding of the complex interplay between SARS-CoV-2 and ocular immunity.

### 8.3. Ocular Immunity and Long-Term Effects of COVID-19

The long-term consequences of COVID-19 on the ocular surface are emerging as a global research priority, with several key areas requiring further exploration. Systematic assessment of the prevalence and impact of chronic ocular conditions, such as dry eye, corneal damage, and conjunctival disease, is essential to understand the long-term effects on visual health [117,118]. Additionally, investigating the interaction between ocular immune responses and systemic inflammation, including the role of cytokine storms in multisystem syndromes, is critical to comprehending COVID-19’s extensive impact [112,119]. The potential ocular side effects of COVID-19 vaccines and antiviral therapies, as well as their effects on modulating immune responses, also warrant in-depth research [120]. Collectively, these studies will establish a solid foundation for improving the long-term management of ocular health in COVID-19 patients.

## 9. Conclusions

SARS-CoV-2 infection has highlighted the importance of the ocular surface as both a potential transmission route and a critical site of immune regulation. This review summarized key mechanisms of interaction between the virus and ocular tissues, including ACE2 and TMPRSS2-mediated viral entry, the roles of innate and adaptive immunity in antiviral defense, and the regulatory roles of cytokines and chemokines in inflammation. It also addressed the long-term impacts of ocular immune responses in COVID-19 patients, such as chronic inflammation and visual impairment.

In clinical practice, early detection and management of ocular symptoms in COVID-19 patients, including screening for viral markers in tears, could support early diagnosis. Targeted treatments, such as cytokine inhibitors or local immune modulators, are recommended to mitigate tissue damage and prevent chronic complications.

From a public health perspective, reinforced protective measures in ophthalmic settings, particularly for high-risk groups and healthcare workers, alongside public education on the role of the ocular surface in viral transmission, are crucial. These strategies will contribute substantially to reducing the global health burden of COVID-19.

## Figures and Tables

**Table 1:** Occurrence, severity, and characteristics of COVID-19–related eye symptoms.

Symptom Type	Occurrence Rate (%)	Severity Classification	Remarks
Conjunctivitis	60–70%	Mild to Moderate	Symptoms are mostly self-limiting and short-term within 7–14 days [4,19].
Dry Eye	20–30%	Moderate to Severe	Associated with tear film disruption [15,20].
Photophobia	10–15%	Moderate	Often accompanied by eye pain and tearing [19].
Blurred Vision	5–10%	Moderate to Severe	Related to corneal or retinal lesions [21,22].
Iritis/Uveitis	1–3%	Severe	Requires immediate treatment to prevent blindness [23,24].

**Table 2:** Unsolved research concerns and priorities in long-term ocular consequences of COVID-19.

Research Concern	Key Hypotheses	Potential Investigative Approaches
Chronic Inflammation	Persistent inflammation may lead to fibrotic changes and corneal opacities, exacerbating vision impairment.	- Longitudinal cohort studies - Biomarkers of inflammation - Histological examination of ocular tissues
Persistent Viral Presence	SARS-CoV-2 may reside within ocular tissues, contributing to chronic disease processes.	- Detection of viral RNA in ocular samples - In vivo imaging (e.g., confocal microscopy) - Analysis of immune response
Sustained Immunological Dysregulation	Dysregulated immune responses may perpetuate inflammation and tissue damage, with potential microvascular complications.	- Immune profiling - Advanced imaging (e.g., OCTA) - Cytokine and chemokine analysis
Microvascular Changes	COVID-19–related microvascular changes may predict irreversible ocular damage.	- OCTA for monitoring vascular changes - Correlation with clinical outcomes
Lack of Long-Term Follow-Up Studies	Limited data on incidence, severity, and progression of post–COVID-19 ocular complications impairs understanding of chronic disease trajectories.	- Establishing multicenter longitudinal studies - Integration of registry databases
Early Detection and Therapeutic Optimization	Identifying early biomarkers and risk factors could reduce the progression of vision-threatening complications.	- Evaluation of imaging markers - Development of tailored therapeutic strategies

## Data Availability

Not applicable.
